# InPBi Quantum Dots for Super-Luminescence Diodes

**DOI:** 10.3390/nano8090705

**Published:** 2018-09-10

**Authors:** Liyao Zhang, Yuxin Song, Qimiao Chen, Zhongyunshen Zhu, Shumin Wang

**Affiliations:** 1 Department of Physics, University of Shanghai for Science and Technology, Shanghai 200093, China; zhangly9@outlook.com; 2 State Key Laboratory of Functional Materials for Informatics, Shanghai Institute of Microsystem and Information Technology, Shanghai 200050, China; phzzys@gmail.com; 3 Key Laboratory of Terahertz Technology, Chinese Academy of Sciences, Shanghai 200050, China; 4 School of Electrical and Electronic Engineering, Nanyang Technological University, Singapore 639798, Singapore; chenqm@ntu.edu.sg; 5 Department of Microtechnology and Nanoscience, Chalmers University of Technology, Göteborg 41296, Sweden

**Keywords:** InPBi, quantum dot, finite element method, super-luminescent diode, emission spectrum

## Abstract

InPBi thin film has shown ultra-broad room temperature photoluminescence, which is promising for applications in super-luminescent diodes (SLDs) but met problems with low light emission efficiency. In this paper, InPBi quantum dot (QD) is proposed to serve as the active material for future InPBi SLDs. The quantum confinement for carriers and reduced spatial size of QD structure can improve light emission efficiently. We employ finite element method to simulate strain distribution inside QDs and use the result as input for calculating electronic properties. We systematically investigate different transitions involving carriers on the band edges and the deep levels as a function of Bi composition and InPBi QD geometry embedded in InAlAs lattice matched to InP. A flat QD shape with a moderate Bi content of a few percent over 3.2% would provide the optimal performance of SLDs with a bright and wide spectrum at a short center wavelength, promising for future optical coherence tomography applications.

## 1. Introduction

Dilute bismide is a new member of the group III-V compound semiconductors and has drawn extensive attention for its potential applications in infrared lasers, solar cells and spintronic devices [1,2,3,4]. Bismuth (Bi) incorporation can introduce bandgap reduction [5,6,7,8,9], increase the spin orbit splitting energy and reduce the temperature sensitivity of the bandgap [10,11]. As one member of the dilute bismides, InPBi was first theoretically predicted in 1988 [12] and experimentally realized in 2013 [13]. InPBi exhibits broad and strong photoluminescence (PL) at room temperature, ranging between 1.4 and 2.7 μm, making it a potential candidate for fabricating super-luminescent diodes (SLDs) applied in optical coherence tomography (OCT). Deep level transient spectroscopy (DLTS) has confirmed that there are two deep levels in InPBi thin film, a P_In_ antisite donor-like level and a Bi-related acceptor-like level [14]. The multiple PL peaks forming a broad spectrum originate from transitions between carriers in conduction and valence bands and the aforementioned deep levels [14]. Attempts to make SLDs out of InPBi thin film and quantum wells were carried out in our group but revealed weak electroluminescence. One probable reason is that the InPBi grown at low temperature was *n*-type with the electron concentration in the order of 10^17^–10^18^ cm^−3^ [15]. For the fabricated *p-n* junction, only a small portion of the depletion region lies in the InPBi layer, subsequently, most of the electron-hole recombination happens out of the InPBi region. Another reason is the weak carrier confinement, in particular for electrons. Recent work has shown that Bi atoms distribute inhomogeneously in the InPBi thin films [16]. There are Bi-rich V-shape nanoscale features at the bottom of the InPBi layer close to the InPBi/InP interface and the PL intensity per thickness varies in the InPBi thin film with the maximum value obtained close to the interface where the nanoscale features are observed. This fact indicates that the major contribution to PL in the InPBi thin film is from the bottom part and it is essential to control this part of InPBi epitaxy with Bi-rich nanostructures.

Quantum dot (QD) is one of the most utilized semiconductor nanostructures which confine both electrons and holes and possess discrete energy levels and density of states. Furthermore, the carrier confinement effect can dramatically enhance the radiative recombination efficiency. Due to the outstanding optical properties, semiconductor QDs have been successfully commercialized in telecom lasers [17], visible wavelength LEDs [18] and so forth. In this work, we propose to use InPBi QDs as the active region for InPBi based SLDs. Thanks to the improved carrier confinement and QD size engineering, the InPBi QD device is expected to outperform the similar device made of InPBi thin layer or quantum wells.

III-V QDs are usually grown by the Stranski-Krastanov (SK) mode, triggered by lattice mismatch [19,20]. However, for the InP_1-x_Bi_x_/InP system with Bi concentration of a few percent, the lattice mismatch is too small to initiate the SK mode growth. Droplet epitaxy was first realized by Koguchi et al. in the early 1990s [21]. It was introduced to grow III-Vs on II-VIs with almost no lattice mismatch. It usually contains two steps: first, deposit of the group III atoms with the absence of the group V atoms, forming group III metal droplets on the surface and second, exposure of the droplets to the group V atoms and crystallization. Droplet epitaxy provides an effective method to experimentally realize InPBi QDs on materials lattice matched to InP substrate.

In this paper, finite element method (FEM) was employed to simulate the strain distribution in InPBi QDs/InAlAs/InP system and calculate energy levels of electrons and holes. First, the strain distribution in InPBi QDs/InP is simulated. The in-plane strain component is then calculated with different Bi contents and geometries of InPBi QDs. Afterwards, In_0.52_Al_0.48_As, lattice matched to InP is used to introduce additional potential barrier for InPBi QDs. The strain distribution of the InPBi QDs/InAlAs structure is simulated and utilized as the input for calculations of the ground states of the electrons and holes. Through controlling the size and composition of InPBi QDs, the ground states of the electrons and holes can be engineered. This work provides a feasible way to fabricate SLDs based on InPBi QDs for the potential application in OCT.

## 2. Methods

**Figure 1:**
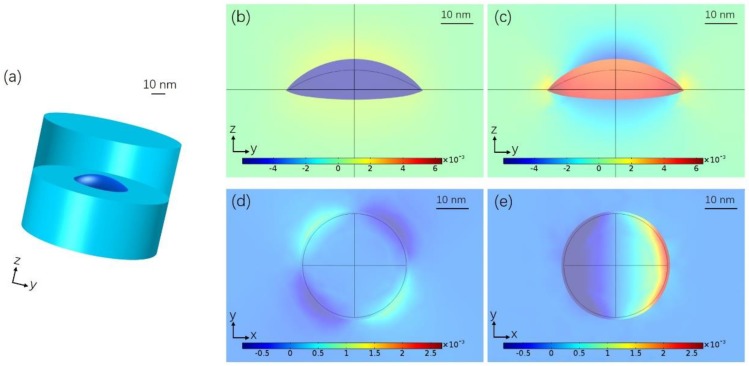
(**a**) Three-dimensional schematic of the proposed InPBi QD structure. The strain distribution of (**b**) *ε_xx_* and (**c**) *ε_zz_* in the *yz* plane; (**d**) *ε_xy_* and (**e**) *ε_xz_* in the *xy* plane for the InPBi QDs in InP with Bi content of 6%, diameter of 40 nm and height of 6 nm. The deformation is exaggerated by 100 times.

Figure 1a shows the schematic of the proposed InPBi QD structure. The InP_1-x_Bi_x_ QD is assumed to be in a spherical crown shape with the height varied from 3 to 20 nm and the diameter varied from 20 to 60 nm, according to the typical III-V QDs grown by droplet epitaxy method. The QD is buried in InP or InAlAs for different calculations. The Bi content (x) is varied from 1% to 12%. FEM was employed to simulate the strain distribution in the InPBi QDs/InP system and calculate the energy levels of electrons and holes in the InPBi QDs/InAlAs structure. The lattice constants of InP (*a*_InP_) and InBi (*a*_InBi_) are 5.87 Å [22] and 6.52 Å [13], respectively, and the lattice constant of InP_1__–_*_x_*Bi*_x_* (*a*_InPBi_) is (1 – *x*)*a*_InP_ + *xa*_InBi_ based on Vegard’s law assumption. The lattice mismatch between InP_1__–_*_x_*Bi*_x_* and InP is:

(1) $$\mathrm{mis}=\frac{a_{\mathrm{InPBi}}-a_{\mathrm{InP}}}{a_{\mathrm{InPBi}}}=\frac{x}{9+x}$$

The elastic coefficients C11, C12 and C44 of InP and InBi are 1011 GPa, 561 GPa, 456 GPa [22] and 60.31 GPa, 32.52 GPa [12], 26.1 GPa [23], respectively. The elastic coefficient of InP_1__−*x*_Bi*_x_* is assumed as:

(2) $$C_{\mathrm{ij}}\left({\mathrm{InP}}_{1-x}{\mathrm{Bi}}_{x}\right)=\left(1-x\right)C_{\mathrm{ij}}\left(\mathrm{InP}\right)+xC_{\mathrm{ij}}\left(\mathrm{InBi}\right)$$

The elastic coefficients C11, C12 and C44 of In_0.52_Al_0.48_As is assumed to be the linear combination of that of InAs and AlAs, which is deduced to be 1033 GPa, 492 GPa and 466 GPa [22], respectively. The conduction band hydrostatic deformation potential *a*_c_ and valence band hydrostatic deformation potential *a*_v_ of InP and In_0.52_Al_0.48_As are −6.0 eV and −0.6 eV [22] and −6.7 eV and −0.8 eV [24], respectively. Due to the lack of available data, the *a*_c_ and *a*_v_ of InPBi are assumed to be equal to that of InP with the consideration that the Bi content is small. Then the ground states of electrons, heavy holes and light holes are calculated by Schrödinger equation with the simulated strain distribution as an input.

(3) $$\left[\frac{-\hbar^{2}}{2m*}\nabla^{2}+V\left(r\right)\right]\Psi \left(r\right)=E\Psi \left(r\right)$$

(4) $$V\left(r\right)=V_{e}\left(r\right)+V_{s}\left(r\right)$$

(5) $$V_{s}\left(r\right)=a_{c,v}\left(\varepsilon_{xx}+\varepsilon_{yy}+\varepsilon_{zz}\right)$$

where *ħ* is the reduced Planck constant and m* is the effective mass of the carriers. For In_0.52_Al_0.48_As, the effective mass of electron ($m_{e}^{*}$) heavy hole ($m_{\mathrm{hh}}^{*}$) and light hole ($m_{\mathrm{lh}}^{*}$) is 0.069*m*_0_, 0.4*m*_0_ and 0.103*m*_0_ [22], respectively, where *m*_0_ is the mass of electron. For InPBi, the effective mass of InP is used instead, that is 0.0795*m*_0_, 0.6*m*_0_ and 0.089*m*_0_ for electron, heavy hole and light hole [22], respectively. *V*(*r*) is the potential, including the potential *V*_e_(*r*) caused by intrinsic band offset and the potential *V*_s_(*r*) introduced by strain. The energy of the InP conduction band minima is set to be 0. Without consideration of strain, the potential *V*_e_(*r*) of the electrons and holes of In_0.52_Al_0.48_As are 0.34 eV and −1.17 eV, respectively. Because the bandgap reduction rate of conduction band and valence band of InPBi is −27 meV/%Bi and 79 meV/%Bi [9], respectively, the potential *V*_e_(*r*) of the electrons and holes of InP_1__–__x_Bi_x_ is −0.027x eV and (−1.42 + 0.079x) eV, respectively. The parameters used for the calculations are summarized in Table 1.

## 3. Results and Discussions

### 3.1. Strain Analysis

At first, strain analysis is carried out for the most simplified prototype model, an InPBi QD buried in InP, as shown in Figure 1a. The simulated InPBi QD has a Bi content of 6%, a diameter of 40 nm and a height of 6 nm. Figure 1b–e shows the distribution of various strain components, *ε_xx_*(b) and *ε_zz_*(c) in the *yz* plane cross the center of the QD and *ε_xy_*(d) and *ε_xz_*(e) in the *xy* plane cross the bottom, respectively. The deformation is exaggerated by 100 times. Because the lattice constant of InPBi is larger than that of InP, the InPBi QD tends to expand, as seen in Figure 1b–e. The *ε_xx_* in the *yz* plane is negative, indicating compressive in-plane strain in the InPBi QD. The *ε_xx_* distributes quite uniformly within the QD with an average value of −5.82 × 10^−3^. The strain in InP around the InPBi QD is tensile and gradually decreases to 0 when it is about 10 nm away from the QD. The *ε_zz_* in the *yz* plane is positive in the InPBi QD, indicating tensile strain in the z direction. *ε_zz_* in the InP above and below the InPBi QD is negative but the strain around the edge of the QD is positive. The shear strain components *ε_xy_* (d) and *ε_xz_* (e) are asymmetric in the *xy* plane. The in-plane strain *ε_xx_*, the vertical strain *ε_zz_* and the shear strain *ε_xz_* are larger than the shear strain *ε_xy_* for about one order of magnitude.

**Figure 2:**
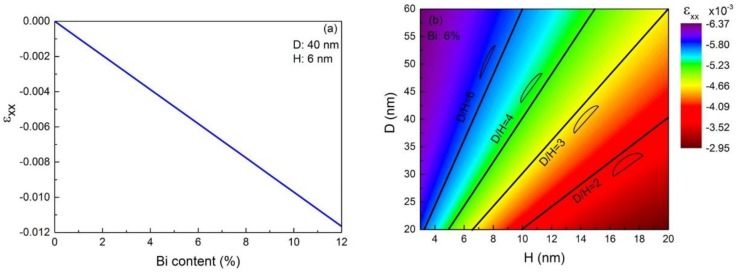
(**a**) The simulated average in-plane strain *ε*_xx_ in the InPBi QDs versus the Bi content with fixed diameter and height of the QDs of 40 nm and 6 nm, respectively; (**b**) contour map of the average *ε*_xx_ versus the diameter and height of the InPBi QDs with the Bi content of 6%. The black lines represent the aspect ratio of 2, 3, 4 and 6, respectively, with the diagrammatic sketch of the shape of the QD next to each line.

Afterwards, the influence of Bi content and geometric parameters on strain distribution is systemically investigated. Figure 2 shows the variation of the in-plane strain *ε*_xx_ versus the Bi content (x) (a) and the aspect ratio (D/H) (b) of the InPBi QD. The *ε*_xx_ shown in Figure 2a is averaged over the whole InPBi QD with a fixed diameter of 40 nm and height of 6 nm, while in Figure 2b the *ε*_xx_ is calculated with Bi content fixed at 6%. The D and H designate the diameter and height of the InPBi QDs, respectively. It can be found in Figure 2a that the average in-plane strain *ε*_xx_ is almost linear to the Bi content with a slope of −0.097. Thus

(6) $$\varepsilon_{xx}=-0.097x$$

The average in-plane strain *ε_xx_* is compressive and its amplitude increases with the Bi content.

Figure 2b is a contour map of the average *ε_xx_* versus the diameter and height of the InPBi QDs together with a few representative lines on which the aspect ratio is the same. The average in-plane strain *ε_xx_* is found proportional to the aspect ratio of the InPBi QDs. The diameter and height of the InPBi QDs vary from 20 nm to 60 nm and 3 nm to 20 nm, respectively, thus, the aspect ratio varies from 1 to 20. The average in-plane strain *ε*_xx_ then varies from −2.95 × 10^−3^ to −6.37 × 10^−3^. For InPBi QDs with different diameters and heights, as long as the aspect ratio is the same, the in-plane strain *ε_xx_* is also the same. The higher D/H ratio, or in another word, the flatter the QD is, the larger the average *ε_xx_*.

### 3.2. Band Structure

In order to enhance the carrier confinement, In_0.52_Al_0.48_As lattice matched to InP is chosen to replace the InP around the InPBi QDs to provide potential barriers for both conduction band and valence band. Thus, the hetero-structure becomes InAlAs/InPBi QDs/InAlAs.

**Figure 3:**
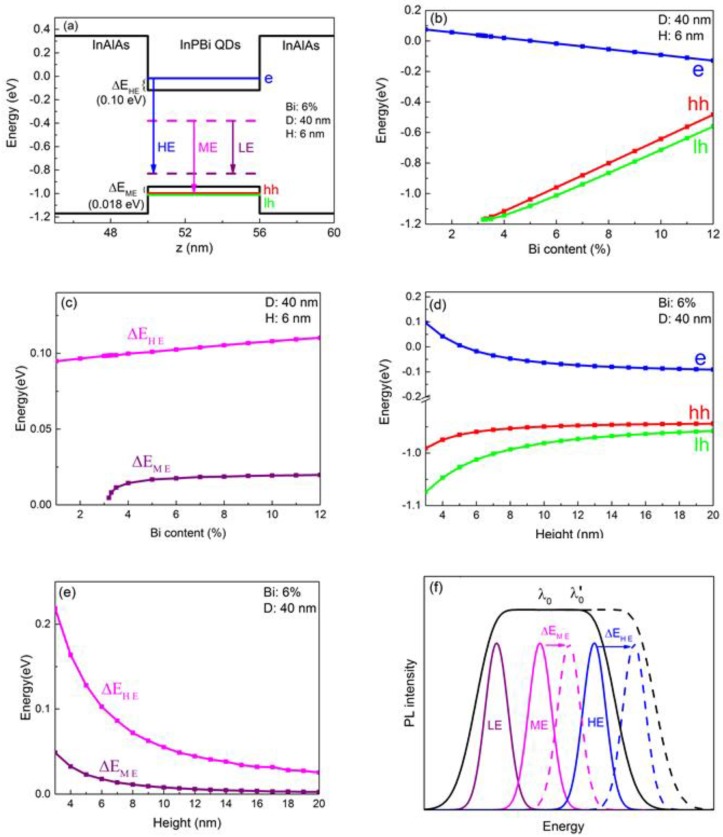
(**a**) the band alignment and carrier recombination processes of an InAlAs/InPBi QD structure plotted along the *z* axis across the center of the InPBi QD with the Bi content, the diameter and the height of 6%, 40 nm and 6 nm, respectively. The zero in Energy is set as the bottom of the conduction band of InP. The blue, red and green line are the ground state of the electrons, heavy holes and light holes, respectively. Δ*E*_HE_ and Δ*E*_ME_ are the energy difference between the electron ground state and the conduction band edge of InP_0.94_Bi_0.06_ and between the valence band edge and the heavy hole ground state, respectively; (**b**,**c**) show the dependence of the ground state energy of the electrons (blue), the heavy holes (red) and the light holes (green) (**b**) and the energy difference Δ*E*_HE_ (magenta) and Δ*E*_ME_ (purple) (**c**) on the Bi content of the InPBi QD with fixed diameter and height of 40 nm and 6 nm, respectively; (**d**,**e**) show the dependence of the ground state energy of the electrons (blue), the heavy holes (red) and the light holes (green) (**d**) and the energy difference Δ*E*_HE_ (magenta) and Δ*E*_ME_ (purple) (**e**) on the height of the InPBi QDs with fixed diameter and Bi content of 40 nm and 6%, respectively. The markers indicate the simulated data points; (**f**) diagrammatic sketch of the PL spectrum broadening with the energy increase of the ME and HE transitions. The solid lines represent the LE (purple), ME (magenta), HE (blue) and PL (black) of the InPBi thin films. The dash lines are the ME transition, ME transition and PL (black) of the InAlAs/InPBi QD structure.

Figure 3a shows the calculated band structure of an InAlAs/InPBi QD/InAlAs hetero-structure, with the diameter, the height and the Bi content of the InPBi QD of 40 nm, 6 nm and 6%, respectively. The bottom of the conduction band and the top of the valence band of InP_0.94_Bi_0.06_ QD lies at −0.12 eV and −0.94 eV, respectively. The blue, red and green lines represent the ground state of the electrons (−0.017 eV), the heavy holes (−1.00 eV) and the light holes (−1.01 eV), respectively. The magenta and purple dashed lines represent the P_In_ antisite level and the Bi-cluster-related level, respectively. According to our former DLTS measurements, there are two deep levels in InPBi thin films. The P_In_ antisite level lies at 0.31 eV below the conduction band of InPBi and the Bi-cluster-related level lies at 0.11 eV above the valence band of InPBi for the InPBi thin film with Bi content of 2.49% [14]. Three radiative recombination processes involving the deep levels were identified from PL measurements. The first is the recombination between the electrons in the conduction band and the holes at the Bi-cluster-related level, marked as HE, the second is between the electrons at the P_In_ antisite level and the holes in the valence band, marked as ME and the third is the recombination between the two deep levels, marked as LE. These three carrier recombination processes together result in very broad PL spectra of InPBi thin films. In the InPBi QD case, due to the quantum confinement, the energy levels of electrons and holes are split into discrete energy levels. Consequently, the recombination with the deep levels will involve the energy levels instead of the band edges of InPBi. The arrows labeled HE, ME and LE in Figure 3a indicate the expected recombination processes in the InPBi QDs. Since the ground state of the heavy holes has lower energy than that of the light holes, the ME process is expected to be between the P_In_ antisite level and the ground state of the heavy holes. The Δ*E*_HE_ and the Δ*E*_ME_ are the energy difference between the electron ground state and the conduction band edge of InP_0.94_Bi_0.06_ and between the valence band edge and the heavy hole ground state, respectively.

The variation of the ground state of the electrons, the heavy holes and the light holes and the energy difference Δ*E*_HE_ and Δ*E*_ME_ with the Bi content and the height of the InPBi QDs are further investigated, as shown in Figure 3b–e. In Figure 3b,c, the diameter and the height of the InPBi QDs are fixed at 40 nm and 6 nm, respectively, while In Figure 3d,e, the diameter and the Bi content of the InPBi QDs are fixed at 40 nm and 6%, respectively. The blue, red, green, magenta and purple curves represent the ground state of electrons, heavy holes and light holes and the energy difference Δ*E*_HE_ and Δ*E*_ME_, respectively. The ground state energy of the electrons is found monotonically decrease with the Bi content varying from 1% to 12%. The absolute value of the ground state energies of the heavy holes and the light holes also monotonically decrease with the Bi content varying from 3.2% to 12%. The difference between the ground state energy of the heavy holes and the light holes increases with the Bi content. When the Bi content is below 3.2%, the valence band edge of InPBi is lower than that of In_0.52_Al_0.48_As and thus there is no potential well for the holes in InPBi, neither the ground states. The dependence of the ground state energies on Bi content is dominant by the bandgap reduction of InPBi. The energy difference Δ*E*_HE_ and Δ*E*_ME_ are found also monotonically increase with the Bi content. The slope of the Δ*E*_HE_–Bi content curve is almost uniform when the Bi content varies from 1% to 12%. However, the Δ*E*_ME_ changes merely when the Bi content is between 4% and 12% and sharply drops when the Bi content decreases below 4%. The dependence of Δ*E*_HE_ and Δ*E*_ME_ on Bi content is mainly caused by the fact that when the Bi content increases, both the conduction and valence band offset increase and the ground states of the carriers are elevated relative to the band edges. The valence band offset is more influenced by the Bi content, even changing from type-I to type-II band alignment at about 3.2%.

Next, the influence of the geometric shape of the QDs on the ground states of the carriers is investigated. The ground state energy levels of the electrons, the heavy holes and the light holes are found to decrease with increasing the height of the InPBi QDs from 3 nm to 20 nm, as shown in Figure 3d. The ground state energy of the electrons remarkably drops when the height of the InPBi QDs increases from 3 nm to 8 nm, resulted from strong quantum confinement when the potential well is thin and then slowly decreases as the height increases from 8 nm to 12 nm. The absolute value of the ground state energy of the heavy holes and the light holes behaves similarly as the electrons. The difference in slopes originates from the difference in effective masses. The energy differences Δ*E*_HE_ and Δ*E*_ME_ in (e) have the same trend as the electron and the heavy hole ground states in (d) since the band edges are fixed in this group of simulations.

Figure 3f is the diagrammatic sketch of the PL spectra of InPBi thin films and QDs. The solid curves represent PL contributions of the LE (purple), ME (magenta), HE (blue) and the overall PL (black) of InPBi thin films, while the dashed curves represent the case of the InAlAs/InPBi QD structure, respectively. Compared to InPBi thin films, the energies of the emitted photons from InPBi QDs are increased with the value of Δ*E*_HE_ and Δ*E*_ME_ for the HE and ME transitions and the center energy of the spectra will also be increased. The high spatial resolution of OCT requires a spectrum with large linewidth and short center wavelength. Based on the above results, InPBi QDs with a low height can increase the Δ*E*_HE_ and Δ*E*_ME_ and thus lead to a wide linewidth as well as a short center wavelength of the emission spectrum, subsequently improving the spatial resolution of OCT.

The ultimate aim of the proposal using InPBi QDs is to produce high performance SLDs with a bright, flat and broad spectrum. The brightness requires a high Bi content to provide large quantum confinement for the carriers. Use of a high Bi content will decrease the bandgap and subsequently decrease the transition energy of HE and ME, leading to a reduced linewidth of the emission spectrum. This can be compensated by controlling the shape of the QDs. Finally, a moderate Bi content of a few percent over 3.2% and a flat QD shape would provide the optimal performance.

Furthermore, unlike the InAs QDs on GaAs platform with large lattice mismatch, the InPBi QDs on InP lattice has limited strain and can thus be stacked for many periods without the risk of strain relaxation. The stacked structure can not only increase the overall intensity but also further engineer the shape and linewidth of the emission spectrum by manipulating the shape and Bi content of the InPBi QDs in each period.

## 4. Conclusions

In this paper, we propose to use InPBi QD as the active region for high performance SLDs. FEM was employed to calculate the strain distribution of the InP/InPBi QDs/InP structure. The in-plane strain components are found larger than the shear strain components in the InPBi QD. The average in-plane strain *ε_xx_* is linear to the Bi content and proportional to the aspect ratio of the InPBi QDs. In_0.52_Al_0.53_As lattice matched to InP is chosen to form potential barriers for the carriers in InPBi QDs. The band alignment and the ground state of electrons, heavy holes and light holes are calculated with different Bi contents and heights of the InPBi QDs. High Bi content can reduce the bandgap and deepen the band offset leading to improved quantum confinement and optical property. Low height can increase both the Δ*E*_HE_ and Δ*E*_ME_, especially the Δ*E*_HE_ and consequently increase the linewidth of the emission spectrum. A moderate Bi content of a few percent over 3.2% and a flat QD shape would provide the optimal performance of SLDs with high light emission efficiency, wide spectrum and shortened center wavelength for future OCT applications.

## Figures and Tables

**Table 1:** Summary of the parameters used for the calculations.

Parameters	InP [22]	InBi	In_0.52_Al_0.48_As
C11 (GPa)	1011	60.31 [12]	1033 [22]
C12 (GPa)	561	32.52 [12]	492 [22]
C44 (GPa)	456	26.1 [23]	466 [22]
*a*_c_ (eV)	−6		−6.7 [24]
*a*_v_ (eV)	−0.6		−0.8 [24]
$m_{e}^{*}$	0.0795*m*_0_		0.069*m*_0_ [22]
$m_{\mathrm{hh}}^{*}$	0.6*m*_0_		0.4*m*_0_ [22]
$m_{\mathrm{lh}}^{*}$	0.089*m*_0_		0.103*m*_0_ [22]
